# Supplementary material for: Ultrafast Backbone Protonation in Channelrhodopsin-1 Captured by Polarization Resolved Fs Vis-pump—IR-Probe Spectroscopy and Computational Methods
Source: Molecules. 2020 Feb 14;25(4):848. doi: 10.3390/molecules25040848 (PMC7070883; doi:10.3390/molecules25040848)
Supplement: Supplementary file 1 [file molecules-25-00848-s001.zip › SI_final 2.pdf]

Ultrafast backbone protonation in channelrhodopsin-1 captured by polarization resolved fs Vis-pump – IR-probe spectroscopy and computational methods.

Till Stensitzki[1], Suliman Adam[2], Ramona Schlesinger[1], Igor Schapiro[2], Karsten Heyne[1]

[1]Institut für Experimentalphysik, Freie Universität Berlin, Arnimallee 14, 1495 Berlin

[2]Fritz Haber Center for Molecular Dynamics Research, Institute of Chemistry, The Hebrew University of Jerusalem, Israel

S1. Detailed discussion of 1530 cm-1 to 1650 cm^{-1} region

Since isomerization takes place with 100 fs, i.e. faster than the time-resolution of this experiment[2], we don't observe excited state contributions in Figure 2. Hence, all positive signals are due to absorption of the photoproduct and all negative signals are induced by ground-state bleaching. We know from our previous works[1,2] that the dynamics of retinal are described with time-constants of 500 fs and 5 ps. Thus, spectral changes on these time scales indicate retinal contributions while slower spectral changes within tens of ps are an indication for protein contributions.

The position of the retinal modes in *Ca*ChR1 is well known from resonance Raman spectroscopy[3-5], therefore we start by assigning the retinal modes in our transient spectra (Figure 2).

The negative peaks at 1540 cm^{-1} and 1623 cm^{-1} can be attributed to the bleaching of the ground-state all-*trans* retinal C=C stretching vibration and the C=N-D stretching vibration of the deuterated Schiff's base (SB). The assignment is supported by the dichroic ratio of both bands: for each band the ratio is larger than two, indicating a near parallel orientation of the corresponding vibrational tdm to the electronic tdm, in line with previous measurements.[6,7] Additionally, both bands are partially decaying within the first 10 ps, in agreement with our prior studies of the C-C fingerprint region. The peak at 1623 cm^{-1} shows a slower decay as well, likely due to an additional obscured band located at 1627 cm^{-1}, as the neighboring band at 1633 cm^{-1} shows almost identical changes.

The high-frequency edge of the bleaching signal at 1540 cm^{-1} shows additional changes in tens of picoseconds centered at 1552 cm^{-1}, likely caused by an underlying protein contribution from a COO- antisymmetric stretching (as) vibration. On the low-frequency side of the 1540 cm^{-1} signal a positive peak at 1530 cm^{-1} is rising with 5 ps. This peak can be attributed to the 13-*cis* retinal C=C stretching vibration of the P_1 state, as reported in the aforementioned resonance Raman studies[3-5].

A broad, partly structured, positive band appears between 1560 cm^{-1} and 1610 cm^{-1}. The broad band decays partially in 5 ps, which is an indication that retinal modes are contributing in this region. The peak at 1602 cm^{-1} of this band can be attributed to the SB C=N-D vibration in the product state P_1, again following the assignment from previous RR studies. Like its ground-state bleaching band, it follows the retinal dynamics and has a dichroic ratio between 2 and 3, again indicating parallel orientation thus supporting the established assignments. The rest of the region is difficult to assign: There are two smaller negative bands at 1600 cm^{-1} and 1585 cm^{-1}, which are either caused by band shifts or by protonation of COO- asymmetric stretching vibrations from aspartates or glutamates. A band-shift also results in a positive signal next to the negative band. Hence, part of the

broad positive signal could be due to the frequency shift of these two modes. At 1575 cm^{-1} a positive band with a dichroic of ~2 is identifiable (Fig. 2). Since the dichroic ratio is different to the adjacent bleaching peak at 1586 cm^{-1}, it is unlikely that they belong together. Instead this signal could be due to an asymmetric stretching COO- mode of Glu. Further contributors to the signal of the broad band could be C=N stretching vibration of arginine[8,9] in a salt-bridge with a carboxylic acid.

The assignment of the negative band at 1635 cm^{-1} is unclear. It has a slightly smaller strength than the SB stretching mode and has a dichroic ratio of about 1. A similar band is also observed in *Cr*ChR2 and was attributed to an arginine C=N vibration.[10] Ogren proved that protein contributions also appear in 80K-FTIR difference spectra in this region. It cannot stem from a partially deuterated sample, since the C=N-H SB vibration in *Ca*ChR1 is located at 1646 cm^{-1}. Potential contributions from the residues located in the vicinity of the retinal could be tryptophan ring vibrations and arginine C=N stretching vibrations.

S2. Comparison of the measured angles with angles in the homology model.

We calculated the relative angles between the electronic tdm of retinal and the C=O groups in the homology model (see S2). Since ultrafast processes in molecules are typically located around its excitation site and propagate with about five angstroms per picosecond, it is reasonable to expect ultrafast interactions within 350 fs (the system response of the experiment) to be located close to the retinal.[11] Thus, we focused on C=O groups in a 6 Å distance of the retinal C_{14} atom given by the homology model. The calculated angles are presented in Figure S1. Only the calculated relative angles of the backbone C=O group of Glu169 and of the carboxyl C=O group of Glu169 agree within the given error margins with the measured values.

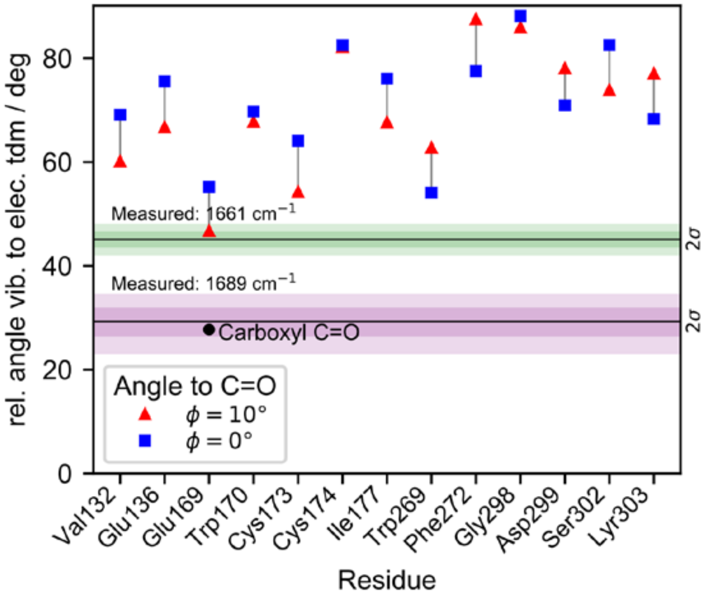

Figure S1. Estimated angles between electronic and vibrational tdms for all amide I vibrations and the carboxylic C=O stretching vibrations in a 6 Å distance of the retinal C_{14} atom given by the homology model. For amide I tdms the estimated angles for 0° (blue squares) and 10° (red triangles) rotation away from the C=O bond in the N-C-O plane are shown. The black circle marks the angle of the one C=O stretching of a carboxyl group in the given volume, the Glu169. The experiment was performed in D_2O where the rotation angle is normally ~10°. The black horizontal lines mark the best fit values of the 1661 cm^{-1} and 1689 cm^{-1} band, with the purple and green shaded ranges marking the 1σ- and 2σ-ranges.

Hse89	87	Val130	79	Tyr167	82	Ile203	83	Leu237	65	Gly279	15
Glu94	72	Tyr131	90	Ser168	84	Ala204	50	Ala238	24	Gln280	34
Ala95	68	Val132	60	Glu169	47	Thr205	48	Ala239	45	Ser281	45
Thr96	36	Cys133	80	Trp170	68	Ile206	73	Lys240	71	Gly282	70
Phe97	54	Cys134	70	Leu171	82	Val207	86	Val241	47	Phe283	52
Ala98	78	Ile135	78	Leu172	68	Phe208	46	Leu242	28	Lys284	84
Hse99	55	Glu136	67	Cys173	54	Gly209	67	Ile243	60	Lys285	78
Val100	28	Leu137	82	Cys174	82	Ile210	89	Ser245	25	Ile286	54
Cys101	66	Val138	82	Pro175	80	Thr211	69	Leu257	72	Ser287	78
Gln102	77	Phe139	61	Val176	65	Ala212	46	Val258	58	Pro288	74
Trp103	42	Ile140	62	Ile177	68	Ala213	78	Lys259	29	Tyr289	42
Ser104	28	Cys141	84	Leu178	72	Met214	85	Ala260	63	Ala290	66
Ile105	79	Phe142	80	Ile179	88	Leu215	44	Met261	76	Asp291	87
Phe106	68	Glu143	52	Hse180	58	Lys220	40	Ala262	49	Val292	60
Ala107	36	Leu144	69	Leu181	88	Ile221	62	Ile263	35	Ile293	38
Val108	57	Tyr145	88	Ser182	69	Ile222	89	Thr264	73	Ala294	71
Cys109	82	Hse146	60	Asn183	31	Phe223	60	Tyr265	78	Ser295	83
Ile110	62	Glu147	50	Val184	46	Tyr224	40	Tyr266	41	Ser296	48
Leu111	35	Phe148	24	Thr185	23	Leu225	75	Val267	41	Phe297	47
Ser112	69	Asp149	79	Gly186	60	Leu226	84	Gly268	71	Gly298	86
Leu113	81	Pro151	40	Tyr191	84	Gly227	42	Trp269	63	Asp299	78
Leu114	49	Cys152	70	Arg194	54	Phe228	60	Ser270	19	Leu300	39
Trp115	44	Ser153	63	Thr195	88	Thr229	85	Phe271	60	Ile301	61
Tyr116	86	Leu154	68	Met196	90	Met230	62	Phe272	87	Ser302	74
Ala117	73	Ile161	79	Gly197	51	Cys231	31	Pro273	45	Asn304	26
Cys125	69	Val162	65	Leu198	70	Cys232	70	Leu274	41	Met305	41
Gly126	69	Asn163	86	Leu199	84	Tyr233	76	Ile275	84	Phe306	83
Trp127	82	Trp164	86	Val200	75	Thr234	49	Phe276	87	Gly307	65
Glu128	62	Leu165	55	Ser201	49	Phe235	47	Leu277	51	Leu308	35
Glu129	74	Arg166	60	Asp202	83	Tyr236	72	Phe278	72	Leu309	63
										Gly310	90
										Hse311	63
										Phe312	42
										Leu313	76

Table S1. Estimated angles between electronic and vibrational tdms for all amide I C=O stretching vibrations in a 20 Å distance of the retinal C_{14} atom given by the homology model.

S3. References

(1) Stensitzki, T.; Yang, Y.; Muders, V.; Schlesinger, R.; Heberle, J.; Heyne, K. Femtosecond Infrared Spectroscopy of Channelrhodopsin-1 Chromophore Isomerization. *Structural Dynamics* **2016**, *3* (4), 043208. https://doi.org/10.1063/1.4948338.

(2) Stensitzki, T.; Muders, V.; Schlesinger, R.; Heberle, J.; Heyne, K. The Primary Photoreaction of Channelrhodopsin-1: Wavelength Dependent Photoreactions Induced by Ground-State Heterogeneity. *Front Mol Biosci* **2015**, *2*. https://doi.org/10.3389/fmolb.2015.00041.

(3) Schnedermann, C.; Muders, V.; Ehrenberg, D.; Schlesinger, R.; Kukura, P.; Heberle, J. Vibronic Dynamics of the Ultrafast All- *Trans* to 13- *Cis* Photoisomerization of Retinal in Channelrhodopsin-1. *Journal of the American Chemical Society* **2016**, *138* (14), 4757–4762. https://doi.org/10.1021/jacs.5b12251.
(4) Muders, V.; Kerruth, S.; Lórenz-Fonfría, V. A.; Bamann, C.; Heberle, J.; Schlesinger, R. Resonance Raman and FTIR Spectroscopic Characterization of the Closed and Open States of Channelrhodopsin-1. *FEBS Letters* **2014**, *588* (14), 2301–2306. https://doi.org/10.1016/j.febslet.2014.05.019.
(5) Ogren, J. I.; Mamaev, S.; Russano, D.; Li, H.; Spudich, J. L.; Rothschild, K. J. Retinal Chromophore Structure and Schiff Base Interactions in Red-Shifted Channelrhodopsin-1 from *Chlamydomonas Augustae*. *Biochemistry* **2014**, *53* (24), 3961–3970. https://doi.org/10.1021/bi500445c.
(6) Nabedryk, E.; Tiede, D. M.; Dutton, P. L.; Breton, J. Conformation and Orientation of the Protein in the Bacterial Photosynthetic Reaction Center. *Biochimica et Biophysica Acta (BBA) - Bioenergetics* **1982**, *682* (2), 273–280. https://doi.org/10.1016/0005-2728(82)90108-6.
(7) Earnest, T. N.; Herzfeld, J.; Rothschild, K. J. Polarized Fourier Transform Infrared Spectroscopy of Bacteriorhodopsin. Transmembrane Alpha Helices Are Resistant to Hydrogen/Deuterium Exchange. *Biophys J* **1990**, *58* (6), 1539–1546.
(8) Huerta-Viga, A.; Amirjalayer, S.; Domingos, S. R.; Meuzelaar, H.; Rupenyan, A.; Woutersen, S. The Structure of Salt Bridges between Arg+ and Glu− in Peptides Investigated with 2D-IR Spectroscopy: Evidence for Two Distinct Hydrogen-Bond Geometries. *J. Chem. Phys.* **2015**, *142* (21), 212444. https://doi.org/10.1063/1.4921064.
(9) Meuzelaar, H.; Vreede, J.; Woutersen, S. Influence of Glu/Arg, Asp/Arg, and Glu/Lys Salt Bridges on α -Helical Stability and Folding Kinetics. *Biophysical Journal* **2016**, *110* (11), 2328–2341. https://doi.org/10.1016/j.bpj.2016.04.015.
(10) Neumann-Verhoefen, M.-K.; Neumann, K.; Bamann, C.; Radu, I.; Heberle, J.; Bamberg, E.; Wachtveitl, J. Ultrafast Infrared Spectroscopy on Channelrhodopsin-2 Reveals Efficient Energy Transfer from the Retinal Chromophore to the Protein. *J. Am. Chem. Soc.* **2013**, *135* (18), 6968–6976. https://doi.org/10.1021/ja400554y.
(11) Qasim, L.N.; Kurnosov, A.; Yue, Y.; Lin, Z.; Burin, A.L.; Rubtsov, I.V. Energy Transport in PEG Oligomers: Contributions of Different Optical Bands. *J. Phys. Chem. C.* **2016**, *120*, 26663-26667.
